# Pericardiectomy for Constrictive and Recurrent Pericarditis: State of the Art Update

**DOI:** 10.1007/s11886-025-02339-z

**Published:** 2026-01-22

**Authors:** Kristina Krzelj, Maria Bakaeen, Tom Kai Ming Wang, Allan Klein, Saberio Lo Presti Vega, Christine Jellis, Deborah Kwon, Michael Tong, Shinya Unai, Marijan Koprivanac

**Affiliations:** 1 Department of Thoracic and Cardiovascular Surgery, Miller Family Heart, Vascular & Thoracic Institute, Cleveland Clinic, Cleveland, OH USA; 2 Department of Cardiovascular Medicine, Miller Family Heart, Vascular & Thoracic Institute, Cleveland Clinic, Cleveland, OH USA

**Keywords:** Pericardiectomy, Recurrent pericarditis, Constrictive pericarditis, Cardiopulmonary bypass

## Abstract

**Purpose of Review:**

This review provides a contemporary surgical view on constrictive and recurrent pericarditis.

**Recent Findings:**

Cardiac magnetic resonance imaging, echocardiography, and heart catheterization are complementary tools for diagnosis and management of patients considered for pericardiectomy. Radical pericardiectomy, unlike total/complete, represents the most extensive resection. Radical or total/complete pericardiectomy via median sternotomy is preferred over partial for both recurrent and constrictive pericarditis, to minimize the risk of symptoms and/or constriction recurrence. Utilization of cardiopulmonary bypass may facilitate the completeness and safety of the procedure. In surgical planning, attention should be paid to intraoperative challenges, especially low cardiac output syndrome, tricuspid regurgitation, and coronary injury. A multidisciplinary approach in experienced centers is essential to optimize outcomes.

**Summary:**

Radical or complete pericardiectomy is advised over partial to avoid the symptoms or constriction recurrence owing to the remaining pericardium. Further research is mandatory to identify the optimal timing and precise extent of pericardiectomy.

## Introduction

Between the publication of the two comprehensive guidelines in the management and treatment of pericardial diseases by the European Society of Cardiology (ESC), 10 years have passed [1, 2]. In that period, numerous studies considering advanced imaging options, and conservative and surgical treatment of pericardial inflammation and constriction have been conducted. This resulted in the recently published American College of Cardiology (ACC) Expert Consensus Statement on the Diagnosis and Management of Pericarditis [3] and the new 2025 ESC Guidelines for the management of pericarditis [2]. The ACC Expert Consensus is a concise clinical guide that focuses on novel approaches in conservative treatment and diagnosis, including the recommendation to perform radical pericardiectomy on cardiopulmonary bypass (CPB), when indicated, in experienced tertiary surgical centers [3]. The new 2025 ESC guidelines represent a more comprehensive document that brings some aspects of the surgical approach to pericardial diseases, including considerations on the extent of resection and CPB use in pericardiectomy. Despite these publications, some issues regarding pericardiectomy remained unaddressed. In this review, we present recent updates on the surgical perspective of pericarditis and constriction.

### Pericardial Inflammation and Constriction

Pericarditis spectrum encompasses acute, incessant, recurrent, and chronic pericarditis. The first self-limiting episode of pericarditis lasting 4–6 weeks is considered acute. Episodes of pericarditis lasting > 4–6 weeks represent incessant pericarditis, and those lasting beyond 3 months are considered chronic pericarditis. The cases with relapses after a symptom-free period of 4–6 weeks represent recurrent pericarditis [3, 4]. 

Common etiologies in developed countries include idiopathic/post-viral, infective, autoimmune, neoplastic, iatrogenic (post-surgical/post-interventional, post-radiation), and others (post-myocardial infarction, post-myocarditis, trauma-related, drug-induced, metabolic) [5]. 

Among patients with acute pericarditis, 20–30% will develop recurrent episodes within 18 months despite antiinflammatory therapy during the initial episode.

The pathophysiology of recurrent pericarditis is not entirely elucidated. Several immunological pathways have been hypothesized to contribute to the relapsing nature of the disease [6]. Recent studies have highlighted that recurrent pericarditis is associated with disturbances in innate immunity, with a pivotal role for the NRLP3 inflammasome and its release of IL-1 [7, 8]. These findings contributed to an increased utilization of anti-IL-1 agents (such as anakinra and rilonacept), which play an important role in the conservative management of recurrent pericarditis resistant to colchicine and glucocorticoid-dependent [2, 3, 9–13]. 

Although the IL-1 receptor inhibitors lower the likelihood of recurrence, the high costs and high rate of relapses after treatment discontinuation limit their widespread use [14, 15]. 

Moreover, recurrent pericarditis requires multiple hospitalizations and treatment over the years, leading to a doubling of the health care costs and significantly diminishing the quality of life [16, 17]. 

The constriction may occur after any type of pericarditis. In Western countries, constrictive pericarditis most commonly develops after the idiopathic/post-viral type of pericarditis, whereas tuberculosis remains the leading cause in developing countries [1–3, 18, 19]. It is estimated that among those with idiopathic or post-viral pericarditis, chronic constriction will develop in less than 1% of cases [20]. In patients with tuberculosis pericarditis, transient constriction occurs in 10%, while progression to chronic constrictive pericarditis occurs in 20% to 50% despite being on effective antituberculosis treatment [19]. 

**Fig. 1:**
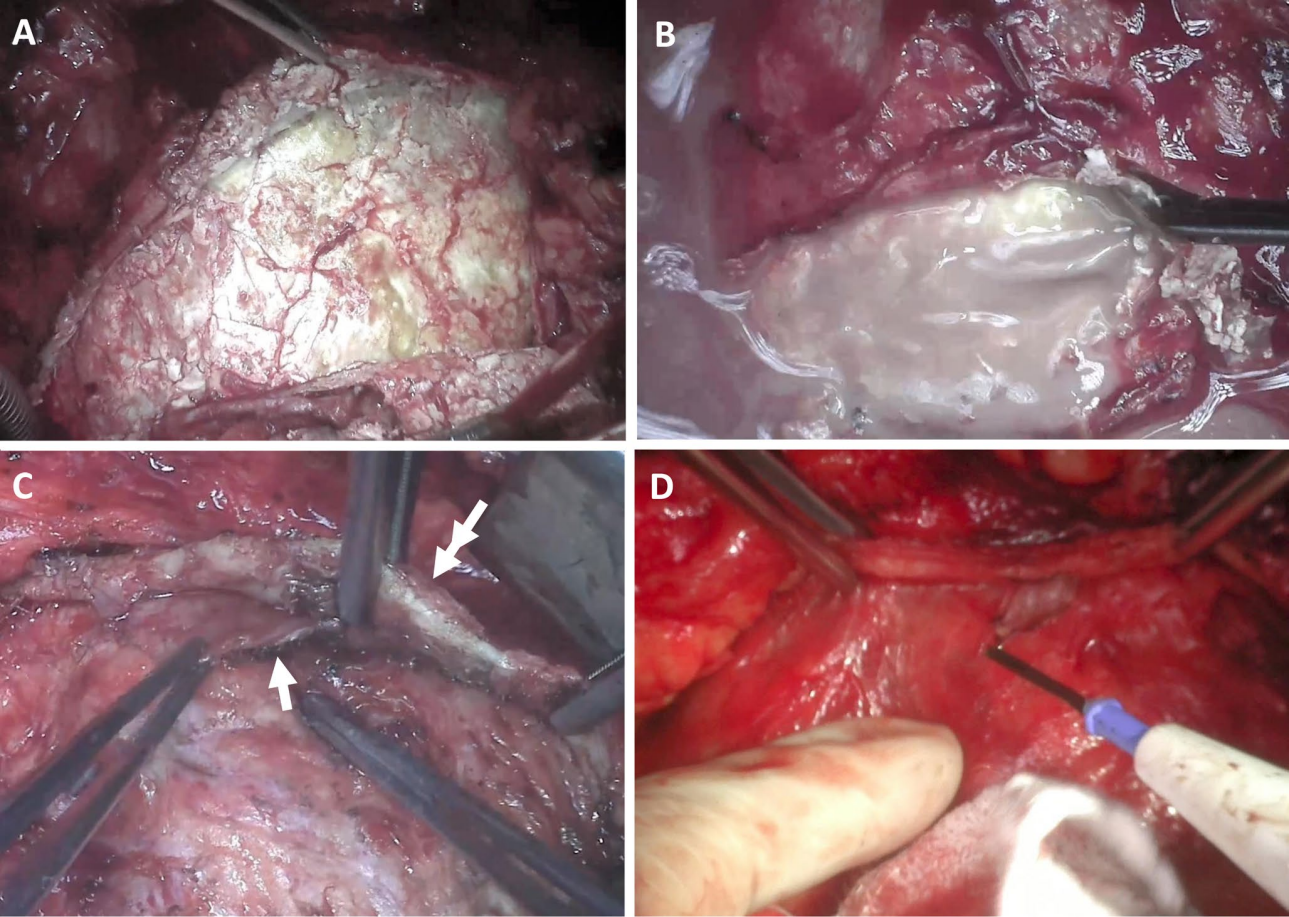
Gross features of **(A)** constrictive pericarditis – calcified pericardium; **(B)** calcific and effusive component of the constrictive pericarditis; **(C)** effusive-constrictive pericarditis –visceral pericardium firmly attached to the surface of the heart (*single arrow*), *double arrow* shows parietal pericardium; **(D)** pericardium in recurrent pericarditis

There are three forms of constriction: transient constriction (a reversible type of constriction caused by pericardial edema associated with inflammation), effusive-constrictive pericarditis (characterized by pericardial effusion and firm attachment of the visceral pericardial layer to the heart surface), and chronic constrictive pericarditis (Fig. 1a-c). Despite the common constriction symptoms, the macroscopic observation of chronic constrictive pericarditis depends on etiology. In idiopathic constrictive pericarditis, the parietal pericardium is significantly thickened and rigid, whereas the visceral layer is discretely inflamed and usually does not interfere with the myocardium. Macroscopic features of tuberculous constrictive pericarditis are caseum and calcified and thickened pericardium, whereas constrictive post-radiation pericarditis is mainly characterized by a firm attachment of the visceral pericardium to the myocardium [21]. In recurrent nonconstrictive pericarditis, the main gross features are fibrin deposits in the pericardial space, focal obliteration of the pericardial space, and confluent adhesions to the myocardium (Fig. 1d). In active pericardial inflammation, the pericardium is thickened and well-vascularized, with a high risk of bleeding during surgery [22]. 

### Diagnosis

Novel diagnostic criteria for pericarditis available in the ACC Expert Consensus and the 2025 ESC Guidelines emphasize the presence of chest pain or equivalent, elevated inflammatory biomarkers, and multimodal cardiac imaging in categorizing the diagnosis of pericarditis into definite, possible, or unlikely [2, 3]. 

The shift towards the increased use of cardiac magnetic resonance (CMR) imaging for the diagnosis of acute pericarditis and evaluating recurrent pericarditis has significantly influenced the tailoring of therapeutic management, severity grading, risk stratification, and decision-making [2, 3, 14, 22, 23]. In patients with recurrent pericarditis who are being considered for pericardiectomy, surgery is not recommended if there is active inflammation observed in CMR imaging. Conversely, a state of minimal inflammation and thus minimal neovascularization of the pericardium on CMR may facilitate the surgical decision to proceed with pericardiectomy, improving the safety and completeness of the procedure [2, 3, 22]. Otherwise, the aggressive medical treatment with anti-IL-1 agents or a gradually decreasing dose of corticosteroids before surgery is recommended in order to minimize the inflammation and ensure better outcomes [2, 3]. On the other hand, a follow-up cardiac MRI is indicated in cases of recurrent symptoms after surgery, particularly in patients with recurrent pericarditis who had significant (moderate to severe) pericardial inflammation prior to surgery, as well as in those with incomplete pericardiectomy. In such cases, follow-up imaging serves to identify a potential residual source of inflammation and provide guidance for subsequent therapy, while its frequency depends on individual symptom burden and response to treatment. Nevertheless, echocardiography is always the first diagnostic method used in cases suspected of pericarditis, and the signs of constriction should always be checked by echocardiography at every recurrence of pericarditis [3, 22]. If constriction signs develop and surgery becomes indicated, a CT scan is required to assess the burden of pericardial calcification and surgical planning.

Right heart catheterization is a valuable complementary tool, not only for delineating constrictive pericarditis from restrictive cardiomyopathy in doubtful cases, but it may be a valuable tool for predicting postoperative outcomes. There are some observations suggesting that the ratio of Right Atrial Pressure / Pulmonary Artery Wedge Pressure RAP/PAWP may be a useful parameter in evaluating myocardial involvement and a predictor of postoperative outcomes. Unlike the patients with heart failure due to myocardial dysfunction, where increased RAP/PAWP was observed as a predictor of mortality [24], in patients with isolated pericardial constriction, higher RAP/PAWP is associated with better long-term survival following pericardiectomy. Hence, RAP/PAWP may be a marker for the identification of patients who may more favorably respond to pericardiectomy [25]. 

## Indications for Pericardiectomy

### Chronic Constrictive Pericarditis

In patients with chronic constriction, pericardiectomy is the cornerstone treatment for most patients with New York Heart Association (NYHA) III and IV status. However, several characteristics should be considered before surgery. Patients with advanced heart failure and cardiogenic cirrhosis (especially with a Child-Pugh Score >7) have little benefit from pericardiectomy, and the operative risk is high [1, 2]. The patients with constriction following radiation usually have the most modest outcomes compared to those with idiopathic or post-interventional/post-surgical pericarditis, most likely owing to radiation-induced cardiomyopathy, coronary disease, and/or pulmonary fibrosis [26, 27]. If chronic constriction is caused by idiopathic/post-viral pericarditis, despite poor preoperative hemodynamic performance, pericardiectomy may bring significant symptom relief and good postoperative short- and long-term survival. It is important to highlight the need for close follow-up of patients after acute pericarditis to recognize the early occurrence of constriction, prevent heart failure development, ensure proper timing of pericardiectomy, and achieve better postoperative outcomes.

### Recurrent Pericarditis Resistant to Medical Treatment

Notwithstanding significant advancements in the medical treatment of pericarditis, up to one-third of patients will experience recurrent symptoms [28]. The recently published ACC Expert Consensus [3] did not elaborate on the precise timing of pericardiectomy in these cases, while the 2025 ESC Guidelines state that pericardiectomy within 6 months following the onset of symptoms is associated with the lowest operative mortality [2]. Moreover, the treatment duration with IL-1 receptor antagonists is also undetermined, although it represents a significant cost and does not bring a definitive solution for some patients with multiple relapses and constriction.

Pericardiectomy may offer successful symptom relief and reduce the number of relapses, with an observed 10-year survival of 80% [29, 30]. Since the observed operative mortality of the pericardiectomy in relapsing pericarditis is low [29, 30], shifting the pericardiectomy as an earlier therapeutic option may be considered as an alternative in younger patients experiencing the most debilitating symptoms at relapses to reduce the number of hospitalizations and improve their quality of life. In these individuals, the number of relapses and severity of symptoms are not the only factors to consider, but also include the side effects of long-term conservative pharmacological management, such as cytopenia, hepatotoxicity, and/or opportunistic infections. However, the postoperative long-term morbidity and mortality in this subset of patients are not well studied. There is only one dedicated study reporting the clinical outcomes after pericardiectomy in recurrent pericarditis thus far conducted [29], which was even before conducted even before the introduction of IL-1 receptor inhibitors in the conservative management. Therefore, in anticipation of new comprehensive guidelines, surgical timing in these patients should be determined by a specialized team [2, 3, 23]. It is also important to continue postoperative follow-up in the same experienced center to ensure strict adherence to postoperative treatment, which includes 3 to 6 months of antiinflammatory therapy with anti-IL-1 agents (anakinra or rilonacept) in patients with active neovascularization revealed in histopathology [2]. Moreover, some patients may still experience recurrent chest pain even after pericardiectomy, especially when tapering antiinflammatory medications. The potential causes of recurrent postoperative chest pain are diverse, such as residual inflammation revealed on CMR, adhesions, incomplete surgical resection, or ongoing autoimmune disease (e.g., rheumatoid arthritis, systemic lupus erythematosus) that require a multidisciplinary approach to optimize patient care.

## Contemporary Approach to Pericardiectomy

### Nomenclature Differences 

Radical, complete, total, and partial pericardiectomy are terms frequently encountered in the literature. However, the variety of terms used for comprehensive pericardiectomy may lead to confusion and should be clearly distinguished. A similar concern could be raised for partial pericardiectomy, as it is unclear to what extent the pericardium is resected. The differences between the terms are summarized in Table 1.

**Table 1:** Terminology and extent of pericardiectomy

Term	Extent of Resection	Key Distinction
**Radical pericardiectomy**	Pericardium completely excised Phrenic nerves skeletonized - left only with fat pedicle, without pericardial strip	Most extensive resection Performed in some high-volume centers as standard of care
**Complete/Total pericardiectomy**	Pericardium resected anteriorly, inferiorly, and variably posteriorly (removed in some centers, preserved in others) Phrenic nerves left with aligning pericardial strip.	Less extensive than radical Terminology varies across institutions
**Partial pericardiectomy**	Resection of anterior pericardium between the phrenic nerves May include removal of diaphragmatic portion of pericardium	Least extensive Extent inconsistently defined across literature

**Fig. 2:**
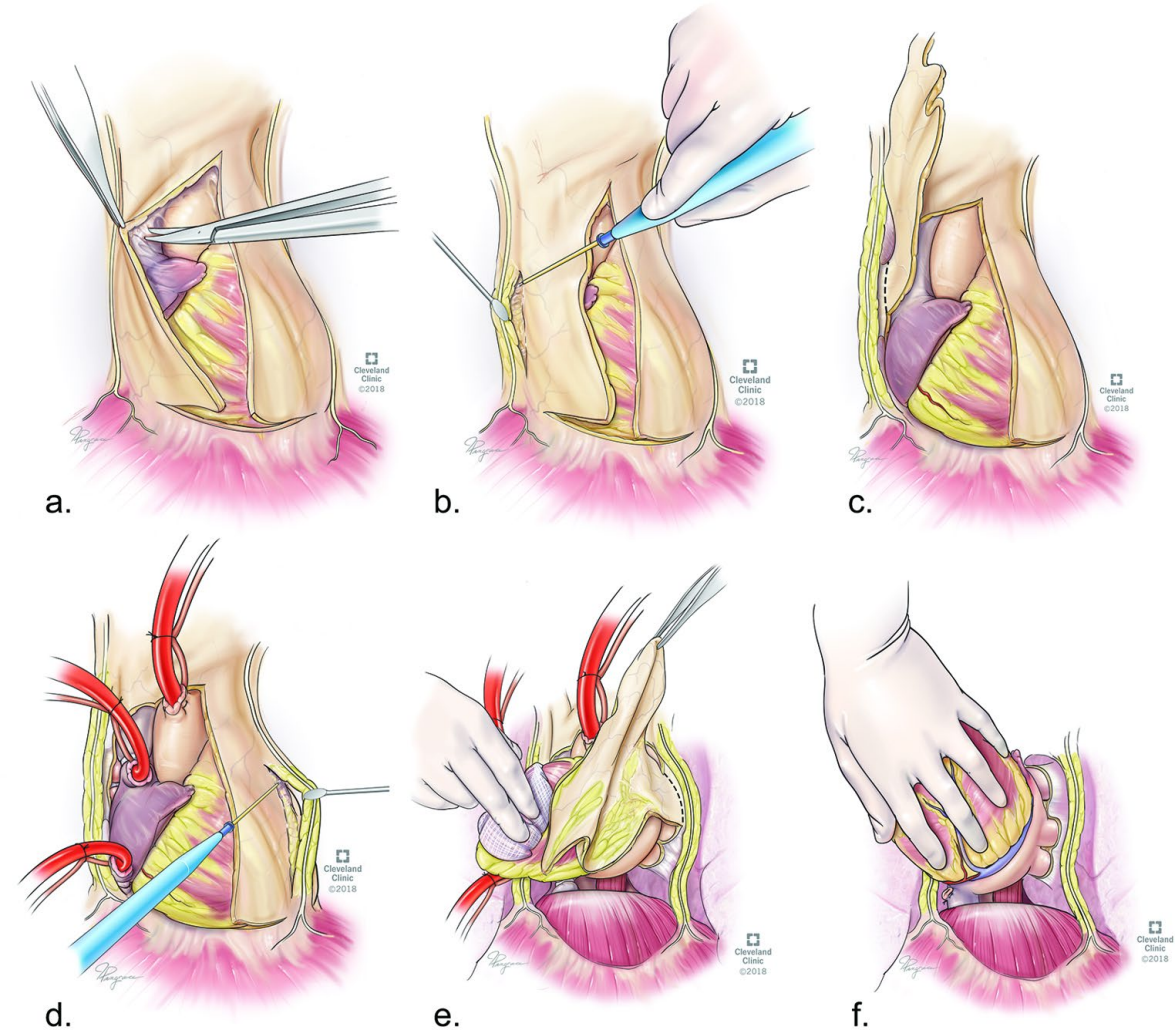
Radical pericardiectomy technique: (**a-d**) removal of the pericardium from the right to the left phrenic nerve, including the skeletonization of both phrenic nerves, (**e-f)** removal of the diaphragmatic and posterior pericardium, including the portion in the oblique sinus and around pulmonary veins. (Reprinted with permission, Cleveland Clinic Foundation ©2025. All Rights Reserved.) (Also used with permission of Elsevier, from: J Am Coll Cardiol, Al-Kazaz M et al., 84(6) ©2024; permission conveyed through Copyright Clearance Center, Inc.) [23]

*Radical pericardiectomy* represents the removal of the pericardium in its entirety, including skeletonization and mobilization of the phrenic nerve, leaving the nerve with its fat pedicle but without the accompanying pericardial strip (Fig. 2). In some pericardial centers, radical pericardiectomy represents the standard of care [23, 31]. Other centers report the use of radical pericardiectomy, but without specifying the details of their resection of the posterior portion of the pericardium and along the phrenic nerves [30]. 

Conversely, some centers adopted complete/total pericardiectomy as their standard. Complete [25, 29, 32–34] or total [35–39] pericardiectomy is less extensive than radical, because it does not include skeletonization of the phrenic nerve. While the pericardium posterior to the left atrium in the oblique sinus is removed in some centers [34–37], is left behind in patients at other centers [26, 32, 33]. 

*Partial pericardiectomy* involves the removal of less than the entire pericardium. Most commonly, it includes the removal of the anterior pericardium from the right to left phrenic nerve, whereas some consider phrenic-to-phrenic pericardiectomy in addition to removing the portion of the diaphragmatic pericardium as partial pericardiectomy.

Nevertheless, in the interpretation of the results from published articles, attention should always be paid to the extent of pericardiectomy that has been performed. The distinction between radical, total, or complete pericardiectomy is especially important in reporting outcomes among patients who undergo pericardiectomy due to recurrent pericarditis, since the pericardial inflammation of the remaining pericardium may cause recurrence of symptoms and need for reoperation. The list of most significant publications on radical and complete/total pericardiectomy is provided in Table 2.

**Table 2:** Major publications on radical and complete/total pericardiectomy (chronological order)

Radical pericardiectomy	Year	Complete/Total pericardiectomy	Year
		Chowdhury UK et al. [41]	**2006**
		Villavicencio MA et al. [32]	**2008**
		Chowdhury UK et al. [37]	**2008**
		Khandaker MH et al. [29]	**2012**
		Cho YH et al. [40]	**2012**
		Cho YH & Schaff HV. [33]	**2013**
		Szabó G et al. [39]	**2013**
		Vistarini N et al. [38]	**2015**
		Busch C et al. [42]	**2015**
Gillaspie EA et al.*[30]	**2016**	Depboylu BC et al. [34]	**2017**
Unai S & Johnston DR. [31]	**2019**	Murashita et al. [26]	**2017**
		Chowdhury UK et al. [35]	**2021**
		Li B et al. [36]	**2024**

*Extent of pericardiectomy reported as radical, but without phrenic nerve skeletonization; resection posterior to the left atrium not specified

### Surgical Approach to Pericardiectomy

From a modern surgical perspective, median sternotomy represents a standard approach to the pericardium, providing excellent exposure of the entire pericardium and ensuring the feasibility and safety of comprehensive pericardiectomy. It is a well-established approach in high-volume pericardial centers of excellence [26, 31]. However, some suggest left modified anterolateral thoracotomy in an off-pump setting as an alternative to median sternotomy in order to achieve radical pericardiectomy, but only in patients with discrete calcifications, excluding those assumed to be technically challenging, those with postradiation pericarditis, patients with recurrent constriction after partial pericardiectomy, or those with previous cardiac surgery [35]. Chowdhury et al. reported their experience with left anterior thoracotomy as an alternative to median sternotomy, which also ensures accomplishment of total pericardiectomy [34]. However, considering the need for CPB and anticipating eventual need for tricuspid valve repair following pericardiectomy, midline sternotomy facilitates cannulation for CPB and allows a fast and safe approach to the tricuspid valve.

### Extent of Pericardiectomy

Following publication of the new 2025 ESC Guidelines and ACC Expert Consensus document, performing partial or radical/complete/total pericardiectomy should no longer be a matter of debate. Previous ESC Guidelines from 2015 advocated a tailored approach for every patient, without providing specific recommendations on the decision-making process for radical/complete/total versus partial pericardiectomy [1]. The recent ACC Expert Consensus Statement recommends radical pericardiectomy for both constrictive and recurrent pericarditis [2] while 2025 ESC Guidelines advocate complete or radical pericardiectomy. However, none of these documents clearly defined the exact extent of radical or complete pericardiectomy, and more evidence is needed to determine whether radical pericardiectomy offers an advantage over total/complete pericardiectomy. Regardless, a clear understanding of the definitions and nomenclature of these surgical strategies is crucial (Table 1).

The advocacy of radical/complete pericardiectomy is based on the increasing number of studies from experienced centers performing rather more radical/complete/total pericardiectomies over partial pericardiectomy [25, 28–30, 33, 34]. This trend stems from observations reporting the recurrence of symptoms and constriction requiring completion pericardiectomy, if partial pericardiectomy was performed at the index operation [30, 39, 40]. These findings were associated with increased mortality rate and heightened operative risk at the redo pericardiectomy [35, 39, 40]. In effusive-constrictive pericarditis, separation of the parietal and visceral pericardium and the firm attachment of the visceral layer to the myocardium may lead to misinterpretation that resection of the parietal pericardium is complete (Fig. 1c), thereby jeopardizing the success of surgery. Conversely, in patients with recurrent pericarditis, the relapsing inflammation of the remaining pericardium is the cause of recurrent, debilitating chest pain.

Thereby, in patients with effusive-constrictive or recurrent pericarditis, total/radical pericardiectomy is essential to minimize the risk of recurrence.

### Cardiopulmonary Bypass Use

The utilization of CPB still sparks debate among surgeons. Historically, CPB was used in a standby fashion for cases where hemodynamic instability impeded the resection, in cases of severe bleeding, and if an additional cardiac procedure was required. This implies the CPB was utilized in cases marked as more severe; therefore, the additional risk of CPB itself in the setting of pericardiectomy was difficult to estimate. However, some studies reported that use of CPB during pericardiectomy does not increase operative risk [37, 38]. When planning pericardiectomy, the advantages of CPB should be acknowledged: Owing to the decompression of the heart, the risk of inadvertent cardiac injury is decreased, it maintains hemodynamic stability and end-organ perfusion; and facilitates completeness of resection, even by less experienced surgeons.

Although some expressed concern about the heightened bleeding risk after on-CPB-performed pericardiectomy [2, 29, 41], the increased use of modern hemostatic tools, such as bipolar irrigated hemostatic sealers [30] and various topical hemostatic agents, has lessened the bleeding control issue. Nonetheless, there is still no consensus regarding the use of CPB, since institutional preferences vary and the conduct of randomized controlled trials to elucidate this question is unlikely. The 2025 ESC guidelines do not universally recommend CPB use, but the advantages of CPB use, especially if the surgery includes excision of the posterior pericardium, or in cases of severe adhesions of the visceral layer to the myocardium, were highlighted [2]. 

### Intraoperative Challenges

Surgical planning of pericardiectomy goes beyond deciding to do partial or radical pericardiectomy, with or without CPB. It is essential to consider all potential obstacles during the procedure.

Firstly, the operating surgeon must be aware of the necessity to remove all pericardial tissues en bloc, or in consecutive steps, especially when parietal and visceral layers are easily separated but with the visceral layer firmly attached to the myocardium, such as in effusive-constrictive pericarditis. As mentioned earlier, any remaining pericardial tissue may cause recurrent constriction and/or inflammation, resulting in relapsing symptoms. This is one of the reasons why patients with an indication for pericardiectomy should be referred to the pericardial disease surgical center of excellence.

Secondly, one of the most common causes of perioperative death after pericardiectomy is low cardiac output syndrome (LCOS), with a reported incidence of 12–28% [30, 42, 43]. Unlike the LCOS associated with other cardiac surgical procedures, which has predominantly an ischemic background, LCOS after pericardiectomy is unique because it is caused by a sudden increase in preload and accompanying overdistension of the right ventricle. At this point, an excessively dilated right ventricle cannot produce adequate ejection, resulting in systemic hypoperfusion. This complication may be fatal, and fluid restriction and low-dose inotropes should be standard to avoid this scenario, with maintaining right atrial pressure <12 mmHg and cardiac index just above preoperative values (1.8-2.2 L/min/m^2^) [23]. Otherwise, mechanical circulatory support may be considered in order to provide additional time for the myocardium to adapt to new preload changes following pericardiectomy [23]. 

Thirdly, moderate or severe tricuspid regurgitation (TR) might appear upon pericardiectomy due to annular dilatation associated with overdistension of the right ventricle (RV). Owing to the increased mortality in those with TR, recent studies suggest performing tricuspid valve repair if the TR is more than mild [26, 44, 45]. Although there are no long-term data on the benefit of this procedure in the context of pericardiectomy, this option seems logical. Since RV failure is the most common cause of LCOS after pericardiectomy, annular stabilization of the tricuspid valve (TV) may prevent further perpetuation of RV failure caused by moderate to severe TR. If TV repair is warranted, there is no observed increase in mortality rate, since the repair itself is a low-risk procedure that does not significantly prolong the duration of the entire procedure [26, 42, 44]. However, the function of the RV should be carefully assessed to avoid catastrophic failure following TV repair in a severely and irreversibly damaged RV.

Fourthly, attention is needed to avoid unintentional coronary artery injury during the procedure, especially in patients with pericardial calcifications extending into the myocardium. Although the feasibility of removing calcified pericardial patches depends on the operator’s experience, in severe myocardial extension of calcified plaques, a small portion of the pericardium may be left in place. Albeit concomitant coronary artery bypass grafting (CABG) may be challenging in patients who undergo pericardiectomy, for planned concomitant CABG in addition to pericardiectomy, there was no significant increase in mortality observed compared to isolated pericardiectomy [46]. 

## Directions for the Future

Firstly, the need for costly targeted antiinflammatory medications, advanced imaging methods for therapy guidance, and technically challenging surgery highlights the importance of treating pericardial disease in high-volume institutions with highly specialized teams. Such an approach ensures the best treatment for these patients and the accumulation of experience and knowledge leading to innovations and improvements in care.

Secondly, given the growing recognition of the potential benefits of surgical treatment in patients with recurrent pericarditis, the limited number of reports on outcomes following pericardiectomy for this indication warrants further research to determine the optimal surgical strategy and its timing. Another issue specific to this subset of patients that should be addressed is whether earlier surgery is advisable due to a prolonged disease course, in order to avoid the long-term side effects of medical treatment (anti-IL-1 agents, immunosuppressants) and to improve quality of life.

Thirdly, an increased recurrence rate of constriction and other pericarditis-related symptoms following partial pericardiectomy, as well as increasing awareness of technical possibilities, should encourage the surgical community to perform radical or complete pericardiectomy to decrease mortality and improve the quality of life of this patient population.

Fourthly, owing to the growing amount of data on novel diagnostic, therapeutic, and surgical approaches to pericardial diseases, there is an urge for new comprehensive guidelines that may contribute to worldwide standardization of the treatment.

## Data Availability

This state-of-the-art review does not contain any new experimental data. All data and findings discussed originate from recent publications, which are available in the cited references.
